# Approaches to Preceramic Polymer Fiber Fabrication and On-Demand Applications

**DOI:** 10.3390/ma15134546

**Published:** 2022-06-28

**Authors:** Soorya Kizhakke Veettil, Ravichandran H. Kollarigowda, Pankaj Thakur

**Affiliations:** 1 Department of Materials Science and Engineering, University of Arizona, 1235 James E. Rogers Way, Tucson, AZ 85719, USA; sooryak@e-mail.arizona.edu; 2 Medicarbone Inc., 2820 E Fort Lowell Rd, Tucson, AZ 85716, USA; 3 Special Centre for Nanoscience, Jawaharlal Nehru University, New Delhi 110067, India; pankajthakur@mail.jnu.ac.in

**Keywords:** preceramic, polymer fiber, spinning polymer fibers, electrospinning, wet spinning, ceramic fibers, ceramic fibers applications, high-temperature ceramics

## Abstract

The demand for lightweight, high-modulus, and temperature-resistant materials for aerospace and other high-temperature applications has contributed to the development of ceramic fibers that exhibit most of the favorable properties of monolithic ceramics. This review demonstrates preceramic-based polymer fiber spinning and fiber classifications. We discuss different types of fiber spinning and the advantages of each. Tuning the preceramic polymer chemical properties, molar mass, functional chemistry influences, and incorporation with fillers are thoroughly investigated. Further, we present the applications of preceramic-based polymer fibers in different fields including aerospace, biomedical, and sensor applications. This concise review summarizes recent developments in preceramic fiber chemistry and essential applications.

## 1. Introduction

The demand for lightweight, high-modulus, and temperature-resistant materials for aerospace and other high-temperature applications has contributed to the development of ceramic fibers. The ceramic fiber materials demonstrate monolithic characteristics [1,2,3,4]; however, they are somewhat breakable. Developing these materials results in high-performance ceramic fibers that can be produced economically. Fillers, additives, and inorganic polymeric materials are essential to making ceramic fibers. An overview of preceramic fiber materials and fabrication approaches is presented in this section [1,5,6,7].

Ceramic fibers include all non-metallic inorganic fibers (oxide or non-oxide) except for fibers manufactured via solidifying glass melts. Organic, polymeric fiber materials cannot be used in ceramic matrix composites (CMCs), because their degradation temperatures are below 500 °C; neither can conventional glass fibers with melting or softening points below 700 °C be used [8,9,10,11,12,13,14].

A perfect fiber structure cannot be practically obtained during processing; thus, the fiber properties are usually well below the theoretical property values calculated for a perfect design. An important goal of fiber spinning and fiber formation is the minimization of structural imperfections through the optimization of production processes. Highly optimized and sophisticated processes are usually more important in high-performance fiber production than the materials used. Thus, manufacturing processes should be examined for flaws or inconsistencies that may compromise fiber quality.

Manufacturing processes for inorganic fibers can be categorized as indirect or direct. An indirect process involves coating fiber materials to produce fibers or non-ceramic precursor fibers. Inorganic fibers are formed by pyrolysis of an organic template fiber, which is then soaked in preceramic precursor materials, or precursor materials are deposited on its surface. A direct process involves the direct spinning of inorganic precursors (solutions of salt, suns, or molten precursors) into “green fibers”, sometimes using organic polymer additives [15,16,17,18,19,20].

The first step in producing non-oxide ceramic fibers is the synthesis of inorganic polymers which have compositions that are determined by the precursor polymers and lengths that depend on the production process. Precursor polymers of non-oxide ceramic materials, such as polysilanes, polycarbosilanes, polysilazanes, polycarbosilazanes, and polyborosilazanes, are organosilicon compounds [21,22,23,24,25,26]. The ceramic composition is determined by the type of precursor ceramic used. The polymer precursor structure should be carefully constructed to obtain ceramic fibers with the desired properties. The preceramic polymer requirements for a fiber concoction include: (1) appropriate rheology for non-Newtonian flows of materials and appropriate viscosity during rotation; (2) reactivity to fuse the fibers for subsequent pyrolysis; (3) controlled degradation during pyrolysis to prevent disorders of the structure, such as scattered material, and to produce high-density fibers with high ceramic performance; (4) controlled formation of nano- or microstructures. Silicon carbide ceramics and fibers are produced using polysilanes and polycarbosilanes as precursor polymers. Polycarbosilanes are sometimes modified by adding metal-organic compounds and other polymers such as polypropylene or polymethylphenylsiloxane. Supplementing polycarbosilanes with polymethylphenylsiloxane results in oxygen-rich SiC fibers.

SiCN-based ceramics and fibers are produced using polycarbosilazanes as precursor materials; Si_3_N_4_-based materials are made using polysilazanes as precursors. 

Production processes can differ based on the fiber lengths produced. There are production processes for continuous and short fibers with ranges from millimeters to centimeters. Continuous fibers are generated through the conventional spinning approach, and short fibers are spun using fast-rotating discs or blowing air. The fibers produced from non-oxide fibers have better mechanical strength and modulus values than traditional oxide fibers because of their chemical structure, which can switch between polycrystalline or amorphous. Amorphous fibers have lower creep rates than polycrystalline oxide fibers at high temperatures. Thus, non-oxide fibers have application restrictions due to the oxidative degradation [27,28,29]. Fibers with a lower oxygen content have greater oxidation resistance. Silicon-based non-oxide ceramics, such as silicon carbide (SiC), silicon oxycarbide (SiOC), silicon nitride (Si_3_N_4_), and their derivatives (SiCN, SiAlON, etc.), are used in a wide range of applications for their heat resistance, chemical stability, excellent mechanical or electrical properties, and other characteristics [30,31,32,33,34,35,36]. Polymers containing organosilicon have been successfully synthesized and used to produce silicone-based ceramics for more than 55 years [37,38,39]. Production of silicon-based ceramics derived from organosilicon polymers generally includes cross-linking, pyrolysis, and ceramization [40,41,42].

**Figure 1:**
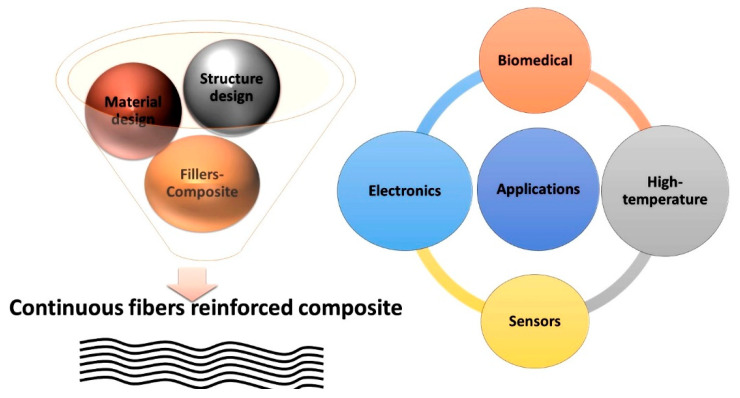
Preceramic polymer fiber fabrication methods and their applications.

Preceramic polymers with novel properties have been developed in recent decades, laying a foundation for ceramic fiber production. Polymer-derived ceramic (PDC) fiber production includes three steps: (i) synthesizing/modifying preceramic polymers; (ii) spinning (melt spinning or electrospinning) and solidifying polymer fibers; (iii) pyrolyzing polymer fibers into ceramic fibers. The combined efforts of chemists, materials scientists, and engineers have produced ceramic fibers with different properties [43,44,45,46,47,48,49,50]. Ceramic fibers can maintain their mechanical properties up to 2000 °C with excellent process oxidation, corrosion resistance, and nanosized structures. They can be used in traditional CMCs and new applications including energy conversion and storage devices. This review presents an overview of PDC ceramic fiber development and future research. This review discusses the functional chemistry, molecular weight influences, surface modification, and on-demand application of preceramic fibers. The preceramic polymer fiber fabrication methods and application of these fibers are presented in Figure 1. 

## 2. Discussion

### 2.1. Production of Fibers

**Figure 2:**
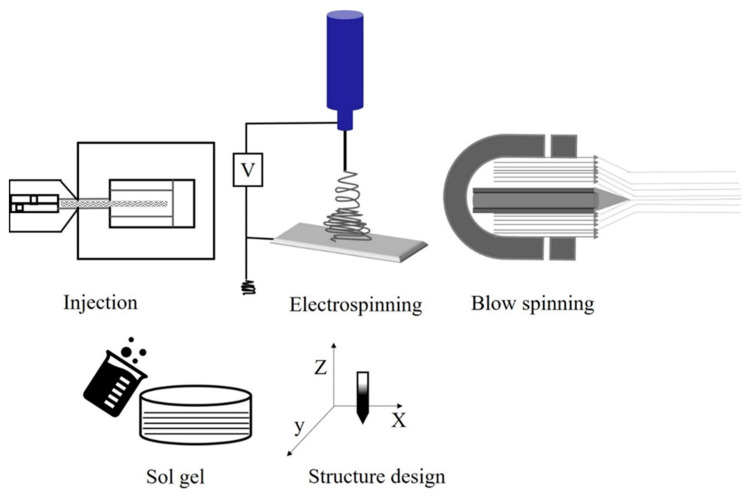
Methods to fabricate ceramic-based polymer fiber structures.

Fiber spinning can be generalized into two primary categories: solution processes, such as dry or wet spinning, and melt spinning. In solution processes, a polymer is dissolved in a solvent to form a “dope” and then extruded through an orifice where fiber consolidation/solidification is triggered through evaporation (i.e., dry process) or coagulation (i.e., wet process). Fiber solidification, in either case, depends on heat and mass transfer; these processes are complex and are used for materials that do not melt/flow easily or that thermally degrade at typical processing temperatures. Consequently, production tends to be slower and costlier. In melt spinning, fiber solidification occurs by cooling the fiber line after extrusion when the polymer is below its melting point; no solvent is present in the polymer melt. Melt-spinning processes are standard industrial processes for many thermoplastics, as they are often the fastest and simplest forming technique for fiber production, relying only on heat transport for solidification [51,52,53,54]. In polymer precursor methods for ceramics, the polymer is shaped using a fiber-spinning method and typically cross-linked before subsequent conversion to a ceramic. Numerous processing methods are available to produce these fibers including thaw spinning [33], sol-gel processes [41,55,56] electrospinning, and blow spinning. An overview of fiber-spinning methods is presented in Figure 2 [41,57].

#### 2.1.1. Electrospinning

Electrospinning is an adaptable and tunable method that uses a simple configuration and process to extract micron to submicron fibers from polymer solutions or molten polymers. All SiC, SiCN, and SiBCN ceramic fibers were once fabricated through melt spinning with a diameter of several microns. Recently, the fabrication of non-oxide ceramic fibers through electrospinning has become popular, as electrospinning can generate polymer fibers with good control of the fiber size and morphology. Similar to melt spinning, appropriate polymer structures are required to obtain uniform electrospun polymer fibers. Theoretical and experimental studies have indicated that the elasticity and entanglement of polymer chains are crucial in obtaining uniform fibers.

Electrospinning has been considered the most promising method for producing continuous fibers. It is also one of the most customizable continuous fiber production methods; the fiber diameter can be adjusted from micrometers to nanometers [58]. In addition, electrospinning can be used to synthesize composite and single-phase fibers. Electrospinning can produce fibers with different composite structure architectures, such as porous core–shell, hollow-shell, stripe-shaped, spongy, core–shell, and hollow structures. There is the possibility of generating nanofibrous layers with a designed aggregate structure including orientation, modeling, and two- and three-dimensional nanonets. In past years, more effort has been invested in producing ceramic fibers using the electrospinning technique [59]. Fibers spun by an electrospinning process are classified according to their structure (micro/nano) and properties, fibers morphology, and chemical structure. These fibers can be used in many applications. The superficial oxide ceramic fibers, such as TiO_2_, Al_2_O_3_, and ZnO, and complex oxide ceramic fibers, such as CaCu_3_Ti_4_O_12_ and Li_1.6_Al_0.6_MnO_4_, are produced by the electrospinning method [59]. Further, ZrC and Cu_2_ZnSnS_4_, which are non-oxide ceramic fibers, are made via the electrospinning process. Table 1 presents several recent simple and complex oxide and non-oxide ceramic fibers produced by electrospinning [60].

Sneddon, Allcock et al. pioneered the production of boron carbide ceramic nanofibers using a single-source polymer precursor polymer from poly(norbornenlyldecaborane) (PND, molecular weight = 32 kDa) [61]. The green boron carbide fibers are drawn through pyrolysis at 1000–1300 °C. SiC electrospinning fibers were drawn by Youngblood et al. using the spinning preceramic polymer of poly(carbomethylsilane) (PCmS). Polystyrene (PS) polymeric-based additives were added to the lower molecular weight of PCmS (3500 Da) polymer solutions to enhance spinnability. Salles et al. describe the submicron-diameter fiber production of novel boron nitride (BN) from a polymer blend solution of polyacrylonitrile (PAN) and poly((B-(methylamino)borazine) [62]. Producing SiCN fibers through electrospinning a commercially available preceramic material, Ceraset™, is a cost-effective fabrication approach. A unique and simple technique has been developed to fabricate Si–C–N–Al ceramic nonwoven mats via electrospinning aluminum-functionalized oligosilazane [63]. Blending preceramic polymers with other commonly used polymers followed by electrospinning can generate nanostructures in polymer fibers through the phase separation between different polymers. This approach allows for the fabrication of ceramic fibers with nanosized structures that can be used in applications where a large surface area is desired. Polyureasilazane (PUS), a low-viscosity thermoset PDC precursor, was blended with poly(methylmethacrylate) (PMMA) [64]. Similarly, polymer-derived silicon oxycarbide (SiCO) ceramic-doped carbon fibers with an average diameter of 165 nm were successfully fabricated using simple electrospinning mixtures of PUS and PAN, a commonly available carbon fiber precursor polymer, followed by oxidative stabilization and pyrolysis at 1000 °C in air and Ar, respectively [65]. Electrospinning of preceramic polymers offers a versatile and controllable approach for producing nanosized ceramic fibers that cannot be achieved by other methods. Integration of other functional materials into polymer fibers introduces additional functionality and structure with possibilities for new applications.

#### 2.1.2. Blow Spinning

The process of producing fibers by blow spinning is straightforward. A homogeneous precursor solution is made by dissolving a polymer in a volatile solvent. A spinnable solution can include different fillers as additives, including metals, ceramics, and metallic salts. The precursor solution is squeezed into a fine needle tip with an internal diameter of 0.1–0.3 mm using a pump. High-speed airflow (10–200 m/s) is provided in the needle direction, and the precursor solution is released as a thin liquid jet. The resulting fiber can be collected as different fibrous materials after evaporation of the solvent from the fiber [66].

In principle, the spinning solution is compatible with blow spinning and does not require a conductive solution. The blow spinning method can be used for most solvable polymers presuming suitable parameters including polymers, solvent, concentration, viscosity, and surface tensions. The preparation parameters, including airflow speed, solution supply speed, diameter of the needle tip, environmental temperature, and relative humidity, must also be considered. The primary steps are selecting a suitable polymer based on the fiber performance and the spinning parameter requirements. Rotta et al. proposed a novel blow spinning technique for fabricating YBCO ceramic nanofibers [67]. The precursor solution was obtained from Y, Ba, and Cu metallic acetates (Ac) and poly(vinyl pyrrolidone) (PVP, Mw = 360,000), with Ac:PVP concentrations of 1:1 and 5:1. Using their novel SBS technique, they obtained a ceramic fiber diameter of 360 nm. A recent article discussing blow spinning techniques included a comprehensive review of ceramic blow spinning [68].

Researchers developing materials for commercial markets can easily adapt the blow spinning process. Safety and scalability are essential for the extensive applicability of nanofibrous material [66] and blow spinning. Solution blow spinning is much faster and more economical than electrospinning for generating nanofibers. Ceramic TiO_2_ and ZnO nanofibers with a large surface area have been fabricated using SBS from a mixture of Ti or Zn sources and polymer in solution, followed by postprocessing [69].

The increased production rate, which increases cost-effectiveness and accessibility to researchers and markets, is a key advantage of blow spinning. Depending on the type of ceramic fiber, production rates typically increase by a factor of 5–10 compared to conventional electrospinning [27,70,71].

In terms of uniformity, electrospun fibers exhibit a more uniform distribution than blow-spun fibers, which form bundles. These fiber bundles significantly impact the fiber’s mechanical properties including rigidity and elasticity. Electrospun fibers can withstand substantially greater stress than blow-spun fibers. Moreover, fiber bundles cause uneven fiber distribution and reduce the specific surface area of the fiber, requiring special attention in manufacturing.

Alternative electrospinning methods and standard industrial-grade techniques have been developed and characterized for specialty applications. These include techniques such as gas jet rotation, centrifugal rotation without nozzles, rotary jet rotation, and rapid rotation. Reviews of fiber-spinning strategies have included brief introductions to SBS or similar techniques [66].

Ceramic nanofibers of TiO_2_ and ZnO with a high surface area have been fabricated using SBS from a mixture of Ti or Zn sources and polymer in solution, followed by postprocessing [72]. The primary advantage of using blow spinning to fabricate these materials is an increased production rate that makes processes more cost-efficient and, thus, more accessible to researchers and markets. Depending on the type of ceramic fiber, production rates typically increase by a factor of 5–10 when using blow spinning compared to conventional electrospinning [67,73].

## 3. Influence of Polymer Structure

**Figure 3:**
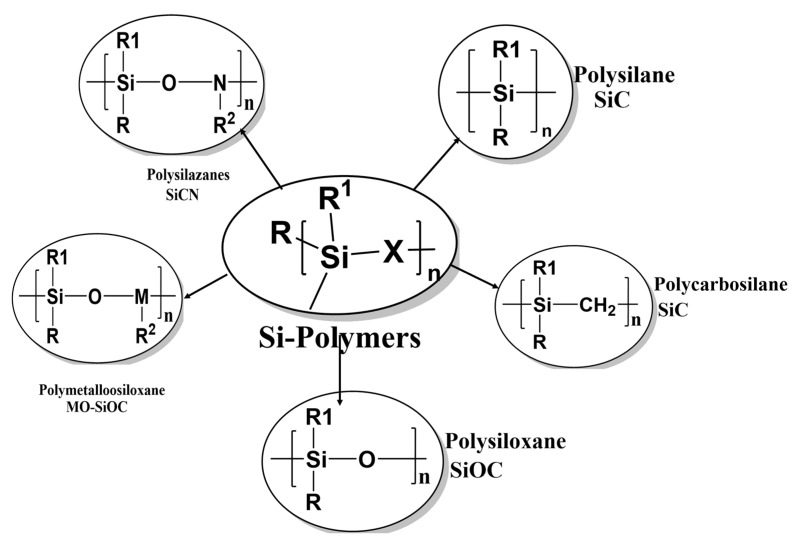
Ceramic conversion of Si-polymers with branched structures.

Branching has a profound effect on the processing of precursor polymers for ceramics. One of the challenges in converting these materials is shape retention during pyrolysis after the initial forming process. Fibers undergo volumetric shrinkage due to the loss of low-molecular-weight volatiles and deformation due to the fact of melting (if semi-crystalline) or creep at high temperatures. Branching significantly increases the molecular weight of the polymer and creates a more amorphous structure that does not melt at high temperatures [74,75]. With increased molecular weight and degree of entanglement due to the change in molecular structure, branched polymers have a higher viscosity that is more amenable to fiber-spinning processes. Laine et al. investigated the effect of linear and star-branched structures on solution-spun polymethylsilane; see the summarized chemical structure in Figure 3. The linear version of the polymer melted and did not have residual reactivity to prevent melting by any other means. 

The star-branched polymer was still spinnable and exhibited superior shape retention [76]. Incorporating small amounts of a known sintering aid (boron) into the polymer backbone, Laine et al. were able to further improve the density of their SiC fiber by reducing grain size and deterring growth in higher temperature processing steps [76]. Branching also increases the ceramic yield [77]. Suttor et al. investigated the effect of branching on cage-structured polysilazanes with molecular weights as high as 800 g/mol. Branching resulted in polymers with increased molecular weight, viscosity, and ceramic yield [77]. 

## 4. Polymer Backbone Structure 

**Figure 4:**
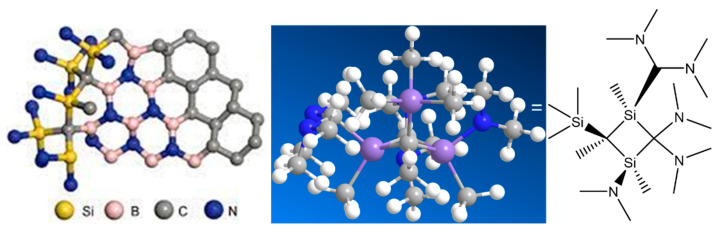
Structure of polymer backbone-derived SiBCN ceramics and functional siloxanes chemistry.

Substitution of polymer structure in BN precursors plays an essential role in creating a melt-spinnable polymer. In BN precursors, cross-linking occurs readily between N-H and B-H couples, forming hydrogen gas. The chemical structure of the backbone is illustrated in Figure 4. However, the cross-linking occurs at low temperatures, preventing melt spinning at higher temperatures. One approach is to reduce the number of reactive sites capable of cross-linking by substituting in the long-chain aliphatic pendant group [78]. This method was reported to help plasticize the polymer melt and reduce cross-linking during processing such that melt-spun fibers could be formed. Unreacted pendant groups (–N(H)CH_3_) in the synthesis of poly(B-(methylamino)borazine) (Duperrier et al.) were thought to help plasticize the polymer (specifically, lowering the glass transition); the best fibers produced from these materials had an intermediate number of –N(H)CH_3_ pendant groups and flexible –N(CH_3_) bridges balanced with cross-links (B-N) between neighboring borazines [79].

The most advantageous feature of polymer-derived multicomponent ceramic fibers is the incorporation of carbon or boron in silica and silicon nitride, which cannot be achieved through synthetic approaches or traditional solid-state reactions. The chemical formulation of silicon nitride and carbon in silica substantially influences ceramic fibers’ chemical structure and properties. Carbon hinders the crystallization of SiO_2_ and Si_3_N_4_ up to 1500 °C. Boron-containing SiCN exhibits more excellent thermal stability and crystallizes at approximately 1700 °C, granting multicomponent ceramic fibers an amorphous matrix for high-temperature applications free of grain-boundary-related properties such as corrosion, grain growth, and creep. In addition, multicomponent ceramic fibers demonstrate excellent tolerance to oxygen, which causes severe degradation of polycrystalline ceramics at high temperatures. The difference in the high-temperature behavior of Nicalon and Hi-Nicalon SiC fibers indicates that eliminating oxygen is crucial for improving creep resistance and crystallization resistance at high temperatures. Pendant groups have also been used to incorporate small amounts of borane into SiC, SiN, and their composites produced through preceramic processes. Wideman et al. added boron-containing pendant groups to a hydridopolysilazane, as boron is known to reduce crystallization in SiC and SiN. Although the addition of borazine resulted in too much residual reactivity for melt-processing, using a monofunctional borane was successful; the material exhibited a greater mass loss during heating but was melt-spinnable [80].

Replacing reactive B-H or N-H groups with a long-chain aliphatic improved spinnability by reducing cross-linking sites (to allow higher temperature processing) and enhanced plasticity.

Xiaoyu Ji recently reported high demand for growing high-temperature structural and functional materials of Si-B–(C)–N ceramic fibers [81]. Hexamethyldisilazane and hexamethyldisilazane polymers are used as co-condensing agents to polymerize silicon and boron chloride monomers, which can tradeoff between spinnability and ceramic yield of polyborosilazanes (PBSZs). The optimal PBSZs can fabricate continuous Si–B–C–N fibers with a homogeneous diameter of 7.9 ± 0.5 μm and a high ceramic yield of 80 wt.%. Mechanical depictions of the influence of pendant groups on polycondensation, melt-spinning, and pyrolysis processes were developed through experimental characterization and quantum chemical computation and improved the understanding of spinnable preceramic polymers in producing high-performance nitride ceramic fibers.

## 5. Interaction with Fillers and Fibers

**Figure 5:**
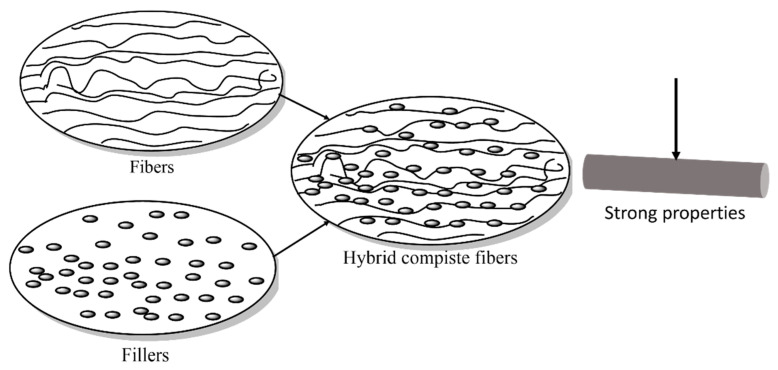
Effect of micro- and nanofillers on the mechanical properties of hybrid nanocomposites.

Composites with fillers or additives often provide better performance than pure polymers. Fillers are classified according to size as macro-, micro-, and nanofillers. Nano-filler-reinforced material offers significantly better performance than micro-and macro-fillers at the same filler loading [82]. How fillers can increase the properties of the materials is illustrated in Figure 5.

Early on, it was recognized that adding suitable filler materials to a preceramic polymer could lead to bulk components that retain their integrity after pyrolysis [83]. Two types of fillers can be used for this purpose: (1) “inert”, or passive fillers, which are ceramic powders that do not react with the ceramic residue from the preceramic polymer, the decomposition gases, or the heating atmosphere, and (2) “active” fillers, which are metallic or intermetallic powders that react during pyrolysis with decomposition gases generated during heating, the heating atmosphere, or (more rarely) with the ceramic residue from the preceramic polymer [84,85,86,87,88].

Mixing preceramic polymers and nanosized active filler particles enables the fabrication of polymer-derived silicate ceramics in different shapes and compositions that can be used in many engineering and specialized applications. The nanosized active filler particles mixed with preceramic polymers facilitate the fabrication of polymer-derived silicate ceramics in different shapes and compositions that can be used in many engineering and specialized applications. Key features of this approach include (a) a low heat required to generate the desired phases, stemming from the high reactivity of the preceramic polymer–ceramic residue with nanosized fillers; (b) suitable mixtures and processing conditions that favor the production of phase-pure components; (c) the possibility of shaping the mixtures into complex 3D bodies using a wide range of plastic-forming and other processing techniques.

Sarker et al. [89] reported that carbon nanotubes (CNTs) as additives were well-dispersed in polyaluminasilazane using a conjugated polymer that increased electrical conductivity 500-fold. The great improvement in electrical conductivity of the fiber samples was attributed to aligned CNTs. The reported method provides a versatile approach for dispersing CNTs in ceramics and a new path to align CNTs in ceramic fibers that may find applications in CMCs with excellent electrical and mechanical properties. Other studies have shown that nanotubes retained their integrity during polymer–ceramic conversion and exhibited strong pullout from the ceramic matrix. Significant improvements in the mechanical properties of the composite, including elastic modulus, hardness, and damage resistance, were observed with the addition of 6.4 vol.% of CNTs.

Mirkhalaf et al. recently reported the compressive results of improving the toughness modulus of polymer-derived ceramics using nanofillers [84]. They used three fillers (i.e., AL_2_O_3_, SiN_4_, and CNTs) and an isostatic pressure of 30 MPa during pyrolysis. They reported that the active fillers, the Al_2_O_3_ or Si_3_N_4_ nanoparticles, were more effective than the nanotubes in improving mechanical properties; 1.5×, 3×, and 2.5× improvements in modulus, hardness, and fracture toughness (JIC) were achieved, respectively. Thus, nanofillers offer significant improvements in composites compared with pure polymers.

## 6. Surface Modification

Modifying the surfaces of ceramic-based materials can modify the properties of the materials. The surface-modified material will enable changes in flexibility and offer hydrophilic (amines, carboxylic acids) and hydrophobic (hydrocarbons or silane-based materials) characteristics.

**Figure 6:**
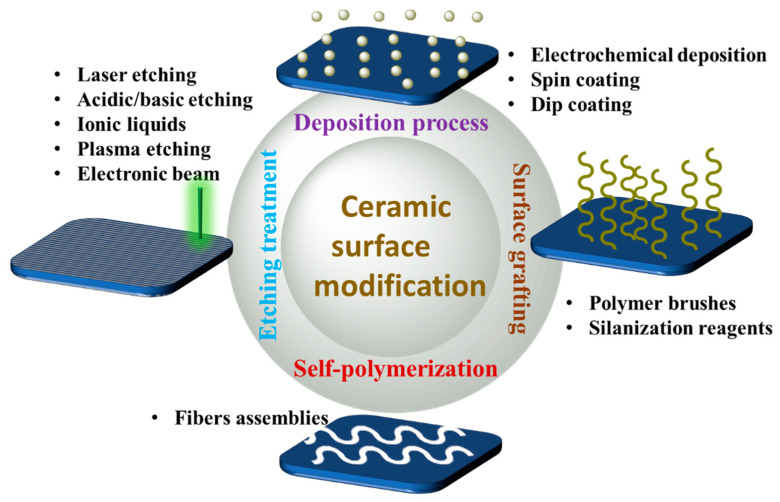
The different methods used for surface modification.

Several methods have been used to modify the surfaces, such as oxidation, etching with ionic liquids, silanization, plasma treatment, and thermal and laser process; see Figure 6. Here, we discuss the two most frequently used surface modification methods. 

*Chemical etching*: Chemical etching is an old method of surface modification method. Several factors are included in this process such as chemical composition, heat, and concentration of reactants in the etching/electrochemical baths. Different reagents can be used for this process including acids and bases [90,91,92].

*Silanization:* Silanization is a process commonly used in current research due to the fact of its many advantages. Surface modification via the silanization method can be performed on powder or solid material. The widely used reagent for the silanization process is organic silane, which can create a monolayer assembly on the surfaces [93,94,95,96,97,98,99,100]. The surface-modified material can enhance and stimulate adhesion to surface/materials. Several commercially available compounds with modified silane surfaces include the functional groups thiol, amine, epoxide, and carboxyl.

*Laser*: Laser surface modification is a unique way of modifying the material’s chemistry, and this process does not make contact with the materials, unlike the silanization and etching process. A process laser can be used as external stimuli to change the surface chemistry remotely without touching and disturbing the material surface [101]. In addition, laser treatment plays a crucial function in supporting surface chemistry. Altering the surface properties can improve the surface wettability of the materials.

*Coating/grafting*: Plasma treatment is the most common process used in industries for the coating/grafting method. Grafting/coating provides bond strength and better mechanical properties, giving good deposition at a low cost. One of the advantages of this coating process is that thickness can be adjustable according to the requirements [102,103].

*Electrochemical deposition*: The electrophoretic deposition process was introduced to simplify the coating method, an alternative to the traditional coating method that is more straightforward and versatile than the conventional coating [104,105]. This method is broadly used to obtain monolayer coatings on ceramic fibers or substrates.

*Self-assembly: Self-assembly* is an autonomous process that organizes a structure or pattern without secondary interference [106]. The monolayer assembly forms naturally upon deposition by spraying active organic compounds onto solid surfaces [107,108]. The main (head) group of the surfactant and the character of the substrate is the main driving force in creating self-assembly. There are three parts in surfactant that help to form an assembly structure: (1) the leading surface group (active head) that attaches to the surface structure; (2) the interfacial properties of the assembly, which are side (terminal) groups; (3) the alkaline chains that link the main and side (head and the terminal) groups. The Van der Waals interface can impact the alkyl chains that orient and stabilize the monolayers. Many factors must be considered when creating a good self-assembly monolayer such as functional chemistry, adhesion and interaction with molecules, solvents, and substrate surface mobility [109,110,111,112].

## 7. Applications of Preceramic Fibers

**Figure 7:**
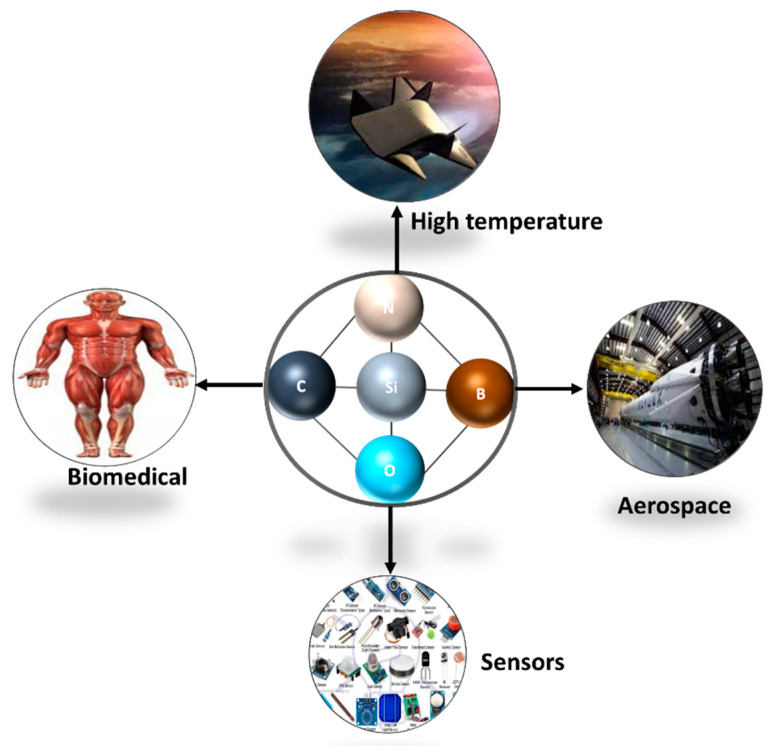
Commercial applications of preceramic fibers.

Ceramic fibers have recently been recognized as innovative materials due to the fact of their unique characteristics and microstructures. Ceramic fibers are used in aerospace, high-temperature material, catalysts, membranes, sensors, biomaterials, fuel cells, parts of electronic devices, and batteries. The potential applications of ceramic fibers are not limited to these; new applications have been introduced in fire-resistant fabrics and sturdy, adsorbent materials. The microstructure, composition, and nanofiber size can be controlled via the spinning procedure; thus, fibers can be developed for specific applications. Several potential applications of ceramic fibers that are discussed in the following sections are shown in Figure 7.

### 7.1. Aerospace

Preceramic-based polymers are useful in aerospace applications for their low-density, high thermal stability, and semiconductor properties. The Micah J. Green group [37,113] analyzed the application and engineering of preceramic polymers for aerospace industries. They showed that preceramic polymers, such as polycarbosilanes, can be heated uniformly and volumetrically using microwaves and radiofrequency waves by adding susceptors such as multiwalled carbon nanotubes. The group has presented a proof-of-concept for radiofrequency and microwave heating methods for SiC fiber processing. 

Rueschhoff [114] recently discussed current information on ceramic materials for aerospace applications, indicating the benefits of ceramic materials including increased temperature capacity, increased erosion resistance, increased rigidity, lower density and, in some cases, multifunctional properties. The addition of additives was also considered. Ceramic additive manufacturing (AM) offers a more agile method for creating complexly shaped components required for next-generation component designs. Additive ceramic and ceramic matrix composites for aerospace industries using direct ink writing were also discussed.

### 7.2. High Temperature 

Materials with stable mechanical and chemical properties up to temperatures slightly below 2000 °C are known as PDCs. They are derived from organic polymer precursors [115]. Over the last decades, high-temperature structural ceramics have gained a reputation as structural materials for their low density, high oxidation and chemical resistance, excellent creep resistance, and thermal shock resistance. The effects of incorporating TiO_2_, HfO_2_, ZrO_2_, Al, and Zr on microstructural and thermal stability have been demonstrated.

Ji et al. investigated the antioxidation of amorphous SiBCN high-temperature fibers [115]. They reported that the SiBCN-based fibers are corroded at 1400 °C in the air and simulated combustion environments. The structural evolution of fibers after corrosion was considered with two conditions and potential mechanisms. 

In “High-Temperature Properties and Applications of Si-based Polymer-Derived Ceramics: A Review”, Ren et al. [116] summarized preceramic-based polymer properties for high-temperature applications. The article provided a thorough discussion of polymer-derived ceramics properties and their influence on microstructure [117].

### 7.3. Biomedical

There have been many efforts to use ceramic nanoparticles (NPs) and nanofibers (NFs) for biomedical applications. The spinning method can fabricate composite ceramic fibers suitable for tissue engineering and other biomedical applications. The benefit of ceramic elements in biomedical applications is their ability to change surface functionality, biomolecular affinity, and other properties. In terms of biomolecular affinity, a greater positive surface electric charge causes more adsorption of negative biomolecules [118]. For example, the unique antimicrobial properties and protein release mechanisms of SiO_2_ have made electrospun polymer-silicate hybrid NFs a candidate for wound-dressing applications [95]. Kurtycz et al. found that a PLA/Al_2_O_3_ NFs mat is nontoxic through indirect cytotoxicity evaluation with human skin fibroblasts [118]. Mahalingam, Alizadeh-Osgouei, and Esfahani summarized recent biomedical applications, including tissue engineering, wound healing, growth factor changes, nerve, vascular grafts, and cardiomyocytes [119,120].

### 7.4. Sensors

Ceramic fibers have an open structure that provides great mechanical strength and good porosity, which allows for the packing of nano/micro functionalized elements on ceramic fibers to encourage their sensing capability in wastewater, medicine, gas treatment, air filtration, and other applications. Several studies have reported ceramic fibers as biosensors. Stafiniak et al. evaluated an electrospun ZnO NF biosensor using a novel method based on standard microelectronic device technology [121]. They reported that the reversible response to physiologically relevant bovine serum albumin (BSA) concentrations in an aqueous solution reached a high sensor current. In another study, the electrochemical reaction of an LaMnO_3_ fiber-modified carbon paste electrode (LaMnO_3_/CPE) for fructose determination was evaluated in the 0.4–100 μM range; a low detection limit (0.063 μM) was observed in comparison with other modified electrodes [122].

Another sensing application for electrospun fibers is the detection of heavy metals, including nitrate, carbonate, and other elements in wastewater or air, for online monitoring of pollutants in actual environments [123]. A linear range of 10–500 μg·L^−1^ was obtained for Pb^2+^ and Cd^2+^; the limits of detection were found to be 3.30 μg·L^−1^ and 4.43 μg·L^−1^ for Pb^2+^ and Cd^2+^, respectively. Hollow ZnO NFs were investigated as an explosive nitro-compound sensor; these NFs were found to sense nitro compounds successfully. However, the sensing performance was greatly affected by the molecular structure of the nitro compounds [60]. 

Kang et al. [124] recently demonstrated the fabrication of a Tm^3+^-doped tellurate glass–ceramic (GC) fiber, enabling enhanced lasing action at ≈2 µm. Compared to as-prepared fibers, the optical conversion efficiency of the GC fiber was increased by 8.8–14.1%. Microstructural analyses and spectral characterization indicated that the laser performance improvement in GC fibers was caused by a significant changes in the local environment surrounding Tm^3+^ due to the formation of nanocrystals within the glass fibers.

### 7.5. Electronic Devices

Ceramic fiber characterization confirms the microstructure, composition, and morphology of a homogeneous compact film as required for photovoltaic cell production. In recent research, Ghashghaie et al. [125] found that electrospun ZnO nanofibers can assemble into the interelectrode space and exhibit dielectrophoresis forces above 1 kHz (5 kHz and 20 kHz) frequencies. The ZnO nanofibers were observed to be aligned along the electric field lines, indicating desirable conditions for electronic device applications [125].

In the future, next-generation wearable textiles will contain nanofibrous membranes capable of converting human biomechanical energy into electricity. Some efforts are under development to construct bioelectric nanogenerators that use nanofibers. Li et al. [126] demonstrated that ceramic fibrous membranes could be tailored to enhance bioelectric nanogenerator polarity, mechanical strength, and surface hydrophobicity, which could eventually improve device performance, power, and capability, even in high-humidity conditions. Wu et al. [127] synthesized a textile with parallel lead zirconate titanate (PZT) fibers through electrospinning that can be used to create flexible and wearable nanogenerators. A comprehensive review of ceramic polymers based on fibers for electronic device applications was recently published [128].

## 8. Conclusions

In this review, we discussed preceramic-based polymer fiber spinning and its classifications. We also discussed different types of fiber spinning and the advantages of each. The properties of preceramic materials, including backbone chemistry, molar masses, pendant group chemistry influences, and incorporation with fillers, were discussed comprehensively. We described modified preceramic materials used in current applications. Preceramic polymer fibers have found new uses due to the fact of their strength, high-temperature characteristics, and modified chemistry and have been used in aerospace, biomedical, and sensor applications. We believe that this review can help scientists understand recent research on preceramic polymer fiber fabrication processes and important applications.

## Figures and Tables

**Table 1:** Comparison of electrospinning and blow spinning techniques [68].

Fabrication	Blow Spinning	Electrospinning
diameter	80–1000 nm	10–800 nm
mean pore size	6–18 μm	5 μm
porosity	~97%	70%

## Data Availability

Not applicable.
